# Durable and efficient gene silencing in vivo by hit-and-run epigenome editing

**DOI:** 10.1038/s41586-024-07087-8

**Published:** 2024-02-28

**Authors:** Martino Alfredo Cappelluti, Valeria Mollica Poeta, Sara Valsoni, Piergiuseppe Quarato, Simone Merlin, Ivan Merelli, Angelo Lombardo

**Affiliations:** 1 San Raffaele Telethon Institute for Gene Therapy, IRCCS San Raffaele Scientific Institute, Milan, Italy; 2 Department of Health Sciences, Università del Piemonte Orientale, Novara, Italy; 3 Institute for Biomedical Technologies, National Research Council, Segrate, Italy; 4 Vita-Salute San Raffaele University, Milan, Italy

**Keywords:** Targeted gene repair, DNA methylation, Gene silencing

## Abstract

Permanent epigenetic silencing using programmable editors equipped with transcriptional repressors holds great promise for the treatment of human diseases^1–3^. However, to unlock its full therapeutic potential, an experimental confirmation of durable epigenetic silencing after the delivery of transient delivery of editors in vivo is needed. To this end, here we targeted *Pcsk9*, a gene expressed in hepatocytes that is involved in cholesterol homeostasis. In vitro screening of different editor designs indicated that zinc-finger proteins were the best-performing DNA-binding platform for efficient silencing of mouse *Pcsk9*. A single administration of lipid nanoparticles loaded with the editors’ mRNAs almost halved the circulating levels of PCSK9 for nearly one year in mice. Notably, *Pcsk9* silencing and accompanying epigenetic repressive marks also persisted after forced liver regeneration, further corroborating the heritability of the newly installed epigenetic state. Improvements in construct design resulted in the development of an all-in-one configuration that we term evolved engineered transcriptional repressor (EvoETR). This design, which is characterized by a high specificity profile, further reduced the circulating levels of PCSK9 in mice with an efficiency comparable with that obtained through conventional gene editing, but without causing DNA breaks. Our study lays the foundation for the development of in vivo therapeutics that are based on epigenetic silencing.

## Abstract (web summary)

Experiments in mice show that designed epigenome editors that contain domains of transcriptional repressors can enable stable epigenetic silencing of *Pcsk9*, a gene with a role in cholesterol homeostasis, without inducing DNA breaks.

## Main

Epigenome editing is emerging as a promising new strategy for silencing genes without altering their primary DNA sequence^3,4^. In this context, designer editors containing an effector domain (ED) derived from naturally occurring transcriptional repressors are targeted to a pre-selected genomic site by a programmable DNA-binding domain (DBD), such as a catalytically deactivated Cas9 (dCas9)^5,6^, transcription activator-like effectors (TALEs)^7,8^ or zinc-finger proteins (ZFPs)^9–11^. Among the different EDs, those belonging to the Krüppel-associated box (KRAB) family of transcriptional repressors^12^ are of particular interest for epigenetic silencing (epi-silencing). KRAB-based editors can induce robust waves of gene repression across different cell types both in vitro and in vivo through a well-conserved mechanism of recruitment of histone-modifying enzymes, and this makes them attractive tools for clinical testing^13,14^. In somatic cells, however, KRAB-associated histone marks are labile unless continuously deposed by a chromatin-bound repressor^15^. Thus, to support prolonged target-gene repression, KRAB-based editors need to be stably expressed in a cell, a task that is usually accomplished by delivering the editors through viral-derived vectors^13,16^. This approach poses safety concerns for clinical translation, as inserting vectors into the host genome can lead to mutagenesis^17,18^ whereas prolonged expression of editors could promote their off-targeting activity, as previously shown^19^ for CRISPR–Cas9. These issues can be solved by adopting combinations of EDs that synergistically act on multiple epigenetic repressive pathways^15^. To this end, we previously took advantage of key EDs from a repressive complex that permanently silences endogenous retroviruses throughout development and adult life^20^: KRAB, the catalytic domain of the de novo DNA-methyltransferase A (cdDNMT3A) and its inactive cofactor DNMT3-like (DNMT3L). Transient delivery of the corresponding combination of editors, termed engineered transcriptional repressors (ETRs), was associated with efficient, durable and specific epi-silencing of endogenous genes in cell lines and in human primary T cells^15^. Epi-silencing operates on the promoter–enhancer region of the ETR-targeted gene through the concerted removal and deposition of activating and repressive histone marks, respectively. It is also accompanied by a local increase in the levels of DNA methylation at CpG dinucleotides, a repressive epigenetic mark that can be inherited throughout cell mitosis by the activity of the endogenous methyltransferase DNMT1. This latter process is at the basis of the durability of epi-silencing, making ETR expression necessary in the initial phases of gene repression and then dispensable for its long-term propagation. Studies have confirmed these findings using all-in-one editors containing, in a single molecule, the above-mentioned EDs and a DBD^21,22^. Moreover, it was shown that the vast majority of protein-coding genes are responsive to epi-silencing, a key step towards the clinical application of the epi-silencing technology^22^. However, whether transient ETR expression can install long-lasting gene silencing in vivo remains unknown. Here we tackled this question by targeting the mouse *Pcsk9* gene, the protein product of which controls circulating levels of cholesterol by promoting the degradation of the low-density lipoprotein (LDL) receptor on the plasma membrane of hepatocytes in the liver^23^. For this reason, inactivation of the *Pcsk9* gene and its product are under intense investigation for the treatment of genetic and acquired hypercholesterolaemia^23–25^.

## In vitro selection of *Pcsk9* ETRs

**Fig. 1:**
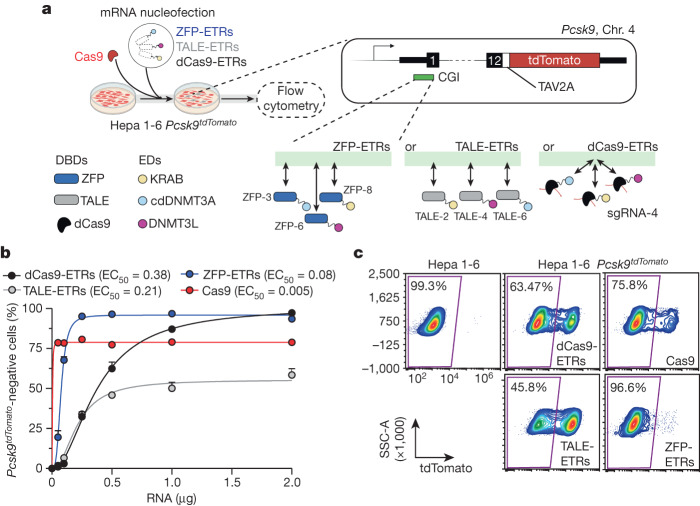
In vitro screen in Hepa 1-6 *Pcsk9*^*tdTomato*^ cells identifies ZFP-based ETRs as the most effective platform for epi-silencing of *Pcsk9*. **a**, Top left, diagram of the experimental procedure used to compare the efficiency of different ETR platforms in the Hepa 1-6 *Pcsk9*^*tdTomato*^ cell line. mRNA nucleofection was used to deliver the ETRs into the cells. As an editing control, cells were co-transfected with mRNA encoding for Cas9 and a gRNA targeting the first exon of *Pcsk9*. Top right, schematic representation of the Hepa 1-6 *Pcsk9*^*tdTomato*^ cell line, in which a TAV2A-tdTomato cassette was targeted in-frame into the last exon of *Pcsk9*. TAV2A denotes a self-cleaving peptide derived from the *Thosea asigna* virus. Bottom right, schematic of the different ETR platforms showing their relative binding to the CGI of *Pcsk9* encompassing its promoter region. Double-headed arrows indicate dynamic binding of the different ETRs to their genomic target sites. The dCas9-based ETRs bind to the same target site, which is dictated by sgRNA-4. Bottom left, key for the pictograms used in the top left diagram. Created with BioRender.com. **b**, Dot plot analysis showing the percentage of *Pcsk9*^*tdTomato*^-negative cells at day 13 after the delivery of ascending doses of mRNAs encoding dCas9-, TALE- and ZFP-based ETRs, and Cas9. Data are mean ± s.d. (*n* = 3). The half-maximum effective concentration (EC_50_) for each editing platform is indicated, as calculated by fitting a four-parameter logistic model (*R*^2^ > 0.98 for all treatments). **c**, Representative flow cytometry dot plots of Hepa 1-6 and Hepa 1-6 *Pcsk9*^*tdTomato*^ cells, the latter analysed at day 29 after RNA nucleofection of the indicated constructs. Data are from Extended Data Fig. 1g. SSC-A, side scatter area. Source data

**Extended Data Fig. 1:**
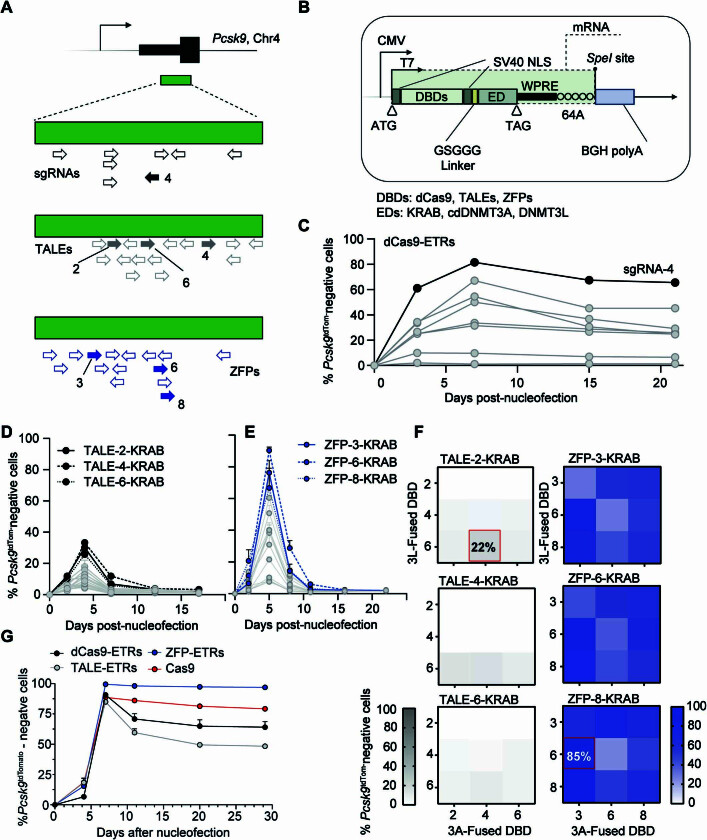
In vitro selection of the most effective ETRs for *Pcsk9* silencing. **a**, Schematic drawing showing on top the *Pcsk9* promoter region with the annotated CpG Island (CGI) and, on the bottom, a zoom on the CGI showing the target sites of all the tested single guide RNAs (sgRNAs; black arrows), TALEs (grey arrows), and ZFPs (blue arrows). Filled arrows indicate the most active sgRNA/DBDs used for subsequent experiments. Created with BioRender.com. **b**, Schematic representation of the plasmid used for ETR expression, either after its direct transfection into cells or as a template for In Vitro Transcription (IVT) of the ETRs’ mRNA. CMV: enhancer/promoter of the Cytomegalovirus. T7: promoter for mRNA production. ATG: start codon; DBDs: DNA-binding domains; SV40 NLS: nuclear localization signal of the simian virus 40; GSGGG: glycine-rich liker peptide; ED: effector domain, either KRAB from the ZNF10 protein, cdDNMT3A or DNMT3L; WPRE: woodchuck hepatitis virus post-transcriptional regulatory element; 64A: stretch of 64 adenines; *Spe*I: restriction site used to linearize the plasmid for IVT; BGH polyA: polyadenylation signal from the bovine growth hormone gene. Created with BioRender.com. **c**, Dot plot showing the percentage of *Pcsk9*^*tdTomato*^-negative cells over a period of 22 days post-delivery of plasmids encoding for the indicated dCas9-based ETRs and 8 different sgRNAs. sgRNA-4 was the most active among the tested guides (black dots and connecting line) and thus used for subsequent experiments. Data are reported as mean (n = 2). **d**, Dot plot showing the percentage of *Pcsk9*^*tdTomato*^-negative cells over a period of 17 days post-delivery of plasmids encoding for 16 different TALE DBDs fused to the KRAB domain. This experiment was meant to identify the most effective TALEs among those tested, using KRAB-mediated epi-silencing of *Pcsk9* as a surrogate readout for DBD efficiency. TALE-2, -4 and -6 were the most active ones among those tested (black dots and connecting line) and thus used for subsequent experiments. Data are reported as mean ± s.d. (n = 4). **e**, Dot plot showing the percentage of *Pcsk9*^*tdTomato*^-negative cells over a period of 22 days post-delivery of plasmids encoding for 16 different ZFP DBDs fused to the KRAB domain. This experiment was meant to identify the most effective ZFPs among those tested, using KRAB-mediated epi-silencing of Pcsk9 as a surrogate readout for DBD efficiency. ZFP-3, -6 and -8 were the most active ones among those tested (blue dots and connecting line) and thus used for subsequent experiments. Data are reported as mean ± s.d. (n = 4). **f**, Left: heat maps showing the percentage of *Pcsk9*^*tdTomato*^-negative cells at day 7 post-delivery of combinations of plasmids encoding for KRAB-, DNMT3L and cDNMT3A-based ETRs containing TALEs. The matrixes were built by transfecting either one of the TALE-KRAB ETRs from **d** with all possible combinations of the three best-performing TALE DBDs (namely, 3, 6 and 8) fused to either DNMT3L (*y* axis) or cdDNMT3A (*x* axis). Given its highest performance, the triple-ETR combination containing TALE-2-KRAB, TALE-6-DNMT3L, and TALE-4-cDNMT3A was chosen for further studies. Colour intensity refers to average silencing efficiency (n = 2). Right: similar experiment as in left but performed with the three best-performing ZFPs from **e**. Given its highest activity, the triple-ETR combination containing ZFP-8-KRAB, ZFP-6-DNMT3L and ZFP-3-cDNMT3A was chosen for further studies. Colour intensity refers to average silencing efficiency (n = 3). Best-performing triple combinations of TALE- and ZFP-ETRs are indicated with a red square. **g**, Time-course analysis of *Pcsk9*^*tdTomato*^-negative cells from the 0.5 µg RNA treatment conditions in Fig. 1b of the main text. Data are reported as mean ± s.d. Source data

To rapidly select for ETR architectures against *Pcsk9*, we developed an engineered mouse hepatoma cell line that reports for the transcriptional activity of this gene at the single-cell level (hereafter Hepa 1-6 *Pcsk9*^*tdTomato*^ cells; Fig. 1a). Using this line, we separately tested and nominated the most effective triple-ETR combination for each of the following programmable DBD platforms targeting the CpG island (CGI) encompassing the promoter region of *Pcsk9*: dCas9, TALEs and ZFPs (Fig. 1a and Extended Data Fig. 1a,b). Specifically, for dCas9-based ETRs, we individually transfected eight single guide RNAs (sgRNAs) together with a previously described triple-ETR combination^15^ in Hepa 1-6 *Pcsk9*^*tdTomato*^ cells and selected sgRNA-4, the one inducing the highest levels of *Pcsk9* repression (Extended Data Fig. 1a,c). For TALE-based ETRs, we followed a sequential approach. First, we fused the KRAB domain to 16 TALEs and identified the top 3 performing ones by measuring *Pcsk9* inhibition at the peak of transient KRAB activity (Extended Data Fig. 1a,d). Then we built ETRs containing KRAB, cdDNMT3A or DNMT3L for each of the selected arrays and delivered all possible permutations of these ETRs as triple combinations in Hepa 1-6 *Pcsk9*^*tdTomato*^ cells. Using efficiency of *Pcsk9* repression as readout, we finally selected TALE-2, TALE-4 and TALE-6 fused to KRAB, cdDNMT3A and DNMT3L, respectively (Extended Data Fig. 1f). A similar selection strategy was used for ZFP-based ETRs, resulting in ZFP-3, ZFP-6 and ZFP-8 fused to cdDNMT3A, DNMT3L and KRAB, respectively (Extended Data Fig. 1a,e,f). On the basis of these data, we conducted a dose–response experiment in the Hepa 1-6 *Pcsk9*^*tdTomato*^ cells by transfecting the RNAs of the selected dCas9-, TALE- and ZFP-based triple-ETR combinations (Fig. 1b). These studies revealed notable pharmacodynamic differences among the three ETR platforms. Specifically, ZFP-based ETRs were 5.7 times and 2.8 times more potent than were dCas9- and TALE-based architectures, respectively, in silencing *Pcsk9* (Fig. 1b). In terms of maximal efficacy, however, both ZFP- and dCas9-based ETRs outperformed TALE-based architectures, reaching at least 80% of *Pcsk9* silencing (Fig. 1b). These latter values were comparable with—if not higher than—those observed after genetic inactivation of *Pcsk9* using matched amounts of RNA encoding for catalytically active CRISPR–Cas9. Gene disruption and epi-silencing of *Pcsk9* proved to be stable until day 28, the last time point analysed (Fig. 1c and Extended Data Fig. 1g). On the basis of these results, we selected the aforementioned ZFP-based ETRs for our subsequent studies.

## Specificity profile of *Pcsk9* ETRs

**Fig. 2:**
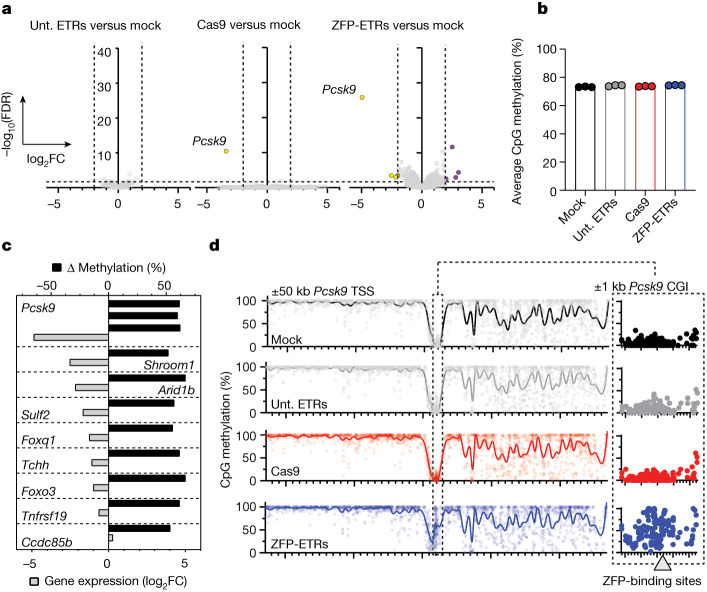
Target-specific transcriptional downregulation with minimal off-target perturbations after epi-silencing of *Pcsk9* by ZFP-based ETRs. **a**, Volcano plots from RNA-seq analyses showing differential gene expression between mock-treated cells and cells treated with untargeted ETRs (unt. ETRs; left), Cas9 (middle) or ZFP-ETRs (right) (*n* = 3 for each experimental condition). The Wald test for binomial distributions was applied for differential gene-expression analysis and *P* values were corrected for multiple testing using the Benjamini–Hochberg approach. The horizontal dashed line indicates the threshold on the adjusted *P* value (FDR ≤ 0.05), and the vertical dashed lines correspond to the threshold on |log_2_FC| ≥ 2. Upregulated genes are in purple and downregulated ones are in yellow. Genes in grey are not differentially expressed according to the applied thresholds. **b**, Bar plot showing the genome-wide levels of CpG methylation of the indicated samples as calculated from the WGMS analyses (*n* = 3 for each experimental condition). **c**, Bar plot showing the correlation between differential methylation and variation in gene expression for the comparison of ZFP-ETRs versus mock. DMRs were associated with a given gene when falling into a ±10-kb window around its own TSS. Plotted are genes for which the |log_2_FC| and FDR can be computed from the differential expression analysis. Black bars indicate the variation in the percentage of CpG methylation of the indicated DMRs. **d**, Left, Manhattan plot from the WGMS in **b** showing the CpG methylation profiles of the indicated samples in a ±50-kb genomic region centred on the TSS of *Pcks9*. Individual dots indicate the average methylation of each CpG. Connecting lines were defined as smoothing spline with 100 knots. Right, magnified view of a ±1-kb region centred on the *Pcsk9* CGI. The genomic region containing the ZFP-binding sites (3, 6 and 8) is indicated in the graph as a grey rectangle. Source data

**Extended Data Fig. 2:**
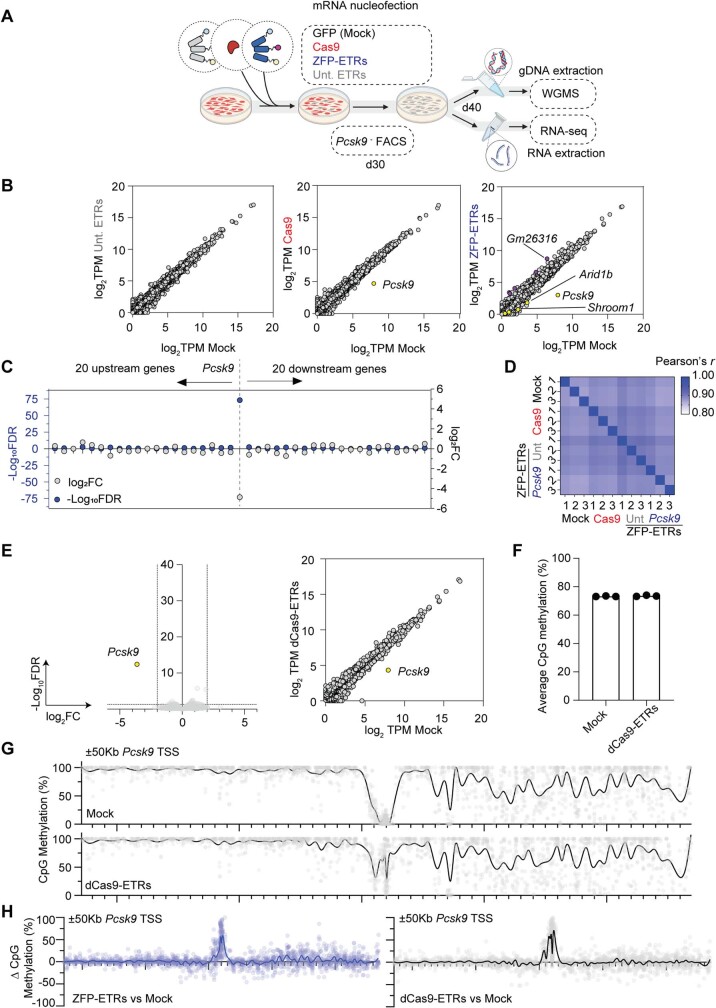
Specificity assessment of ZFP- and dCas9-based ETRs. **a**, Schematic drawing showing experimental procedures for in vitro specificity profile of ETRs. Created with BioRender.com. **b**, Scatter plots from RNA-seq analyses comparing the gene expression levels between mock and either untargeted ETRs (Unt. ETRs; left), Cas9 (middle) or ZFP-ETR (right) treated cells. Purple and yellow dots indicate genes significantly up- and downregulated, respectively; grey dots indicate genes considered not differentially expressed. Thresholds were set at FDR ≤ 0.05 and |log_2_FC| ≥ 2. Data are expressed as log_2_ of transcript count per million (TPM) of mapped reads. **c**, Differential gene expression analysis of 20 genes either up- or downstream of *Pcsk9* from RNA-seq analysis. **d**, Heat map of Pearson’s correlation among WGMS replicates calculated using the cor function after filtering by the coverage, normalizing and considering the positions shared by all the replicates in each condition. Values are reported for each replicate (1, 2 and 3) in each condition. **e**, Volcano plot (right) and scatter plot (left) from RNA-seq analyses showing differential gene expression between mock and dCas9-ETR-treated cells (n = 3). Yellow dots indicate genes significantly downregulated; grey dots indicate genes considered not differentially expressed. Thresholds were set at FDR ≤ 0.05 and |log_2_FC| ≥ 2. For the scatter plot, data are expressed as log_2_ of transcript count per million (TPM) of mapped reads. **f**, Bar plot showing the genome-wide levels of CpG methylation of the indicated samples as calculated from the WGMS analyses (n = 3 technical replicates). **g**, Manhattan plot from WGMS showing the CpG methylation profiles of the indicated samples in a ±50-kb genomic region centred on the TSS of *Pcks9*. Individual dots indicate the average methylation of each CpG. Connecting lines were defined as smoothing spline with 100 knots. **h**, Manhattan plot from WGMS showing differential methylation of CpGs in a ±50-kb genomic region centred on the TSS of *Pcks9* between the indicated samples and mock-treated cells. Individual dots show the differential methylation between the indicated samples at each CpG site. Connecting lines were defined as smoothing spline with 100 knots. Source data

We then assessed the specificity profile of the ZFP-ETRs by transcriptomic and DNA methylation analyses of ETR-treated Hepa 1-6 *Pcsk9*^*tdTomato*^ cells using RNA sequencing (RNA-seq) and whole-genome methylation sequencing (WGMS) (Extended Data Fig. 2a), respectively. Cells transfected with mRNAs encoding for eGFP (hereafter, mock) or for *Pcsk9-*targeted CRISPR–Cas9 were used to control for effects related to treatment and/or *Pcsk9* inactivation. In addition, cells were transfected with a triple-ETR combination equipped with a ZFP array targeting the eGFP sequence (hereafter, untargeted ETRs). When compared to mock-treated cells, no genes were differentially expressed in cells that were treated with untargeted ETRs, whereas both epigenetically silenced and *Pcsk9*-disrupted cells showed a significant reduction (around 30-fold) in the expression levels of *Pcsk9* (Fig. 2a and Extended Data Fig. 2b). In contrast with *Pcsk9*-disrupted cells, in which no other gene was differentially expressed, treatment with ZFP-ETRs caused the deregulation of eight additional genes (four downregulated and four upregulated; Supplementary Table 1), albeit at a lower magnitude than for *Pcsk9*. None of these genes was in the proximity of *Pcsk9*, and none of the 40 genes adjacent to *Pcsk9* showed significant transcriptional deregulation (Extended Data Fig. 2c), indicating that epi-silencing did not spread to nearby genes. In line with the transcriptomic data, the genome-wide levels of CpG methylation were largely superimposable among samples, with a delta methylation of less than 1.2% between ETR- and mock-treated cells (Fig. 2b and Extended Data Fig. 2d). These values are comparable with those previously reported for similar epi-silencing platforms^22,26,27^. In terms of differentially methylated regions (DMRs; delta methylation ≥ 0.4, *P* ≤ 0.001), beyond *Pcsk9*, which contained 3 of them, 18 other DMRs were found in ETR- versus mock-treated cells (Supplementary Table 2). Intersection of transcriptomic and DNA methylation analyses showed that eight out of the nine genes associated with a DMR were also downregulated (Fig. 2c and Supplementary Table 2), with *Pcsk9*, *Shroom1* and *Arid1b* under the selected threshold values (log_2_-transformed fold change (|log_2_FC|) ≥ 2; false discovery rate (FDR) ≤ 0.05). Analysis of the *Pcsk9*-containing locus (±50 kb centred on the transcription start site (TSS) of the gene) at the single-CpG-resolution level confirmed de novo deposition of DNA methylation exclusively around the ETR target site (Fig. 2d and Extended Data Fig. 2h). To understand whether the observed perturbations were due to off-targeting of the EDs, we performed RNA-seq and WGMS of Hepa 1-6 *Pcsk9*^*tdTomato*^ cells treated with the triple dCas9-based ETR combination targeting *Pcsk9*. In contrast with *Pcsk9*, which was robustly downregulated and whose regulatory sequences were de novo methylated in dCas9-ETR-treated cells, no other genes were significantly deregulated and no DMRs were detected (Fig. 2a,b,d and Extended Data Fig. 2e–h), consistent with the data obtained with untargeted ETRs (Fig. 2a,b,d). Overall, these data show that treatment with ZFP-based ETRs imposes limited perturbations outside of the *Pcsk9* gene, and that these variations are likely to be due to mismatched binding of the ZFP arrays at potential off-target sites.

## Durable epi-silencing of *Pcsk9* in vivo

**Fig. 3:**
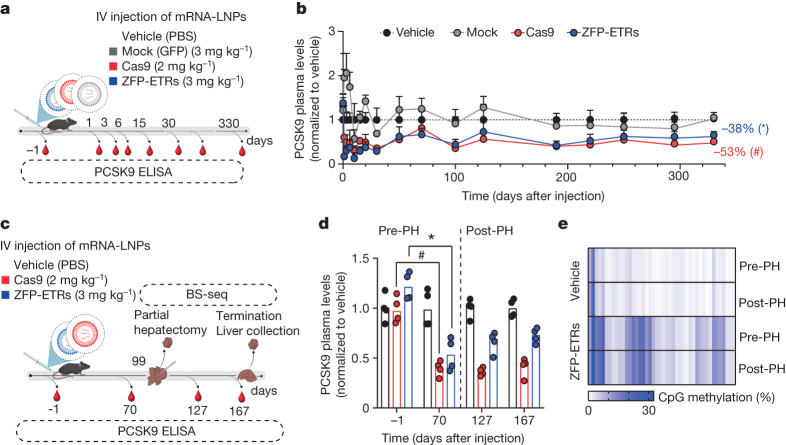
Durable epigenetic silencing of *Pcsk9* in mouse liver after LNP-mediated delivery of ZFP-ETRs. **a**, Drawing of the experimental procedure that was used to assess the efficacy and durability of *Pcsk9* editing in vivo. LNP D was loaded with mRNAs of ZFP-ETRs, Cas9 or eGFP and injected intravenously (IV). Before and after LNP injection, blood samples were collected to measure the levels of PCSK9 by ELISA. LNP doses are indicated as milligrams per kilogram. Vehicle, PBS-treated mice. Created with BioRender.com. **b**, Time-course analysis of circulating PCSK9 levels for up to 330 days after LNP injection. Data are mean ± s.d. (*n* = 7 for ZFP-ETR-treated, 3 for Cas9-treated, 5 for mock-treated and 4 for vehicle-injected mice). Statistical analysis by two-way repeated-measures (RM) ANOVA and Dunnett’s multiple comparisons test between vehicle and the other treatment conditions at the latest time point of analysis (*P = 0.0003 and #*P* = 0.0272). If not indicated, differences were not statistically significant. **c**, Drawing of the experimental procedure that was used to assess editing durability after partial hepatectomy. BS-seq, targeted bisulfite sequencing. Created with BioRender.com. **d**, Bar plot showing the levels of PCSK9 before and after partial hepatectomy (PH). Data for individual mice are normalized to the mean of vehicle-treated mice (dots); bars indicate the median (*n* = 4 for each experimental group). Statistical analysis by two-way RM ANOVA and Dunnett’s multiple comparisons test was performed among samples belonging to the same treatment (mock, ZFP-ETRs and Cas9) at different times (*P = 0.0148 and #*P* = 0.0040). If not indicated, differences were not statistically significant. **e**, Heat map showing the average methylation at single-CpG resolution within the *Pcsk9* CGI in treated (ZFP-ETRs) and control (vehicle) samples before and after the partial hepatectomy. Colour intensity refers to the percentage of CpG methylation (mean of *n* = 4 for each experimental group). Each rectangle represents an individual CpG in the genomic region Chr. 4: 106,463,706–106,464,363. Source data

**Extended Data Fig. 3:**
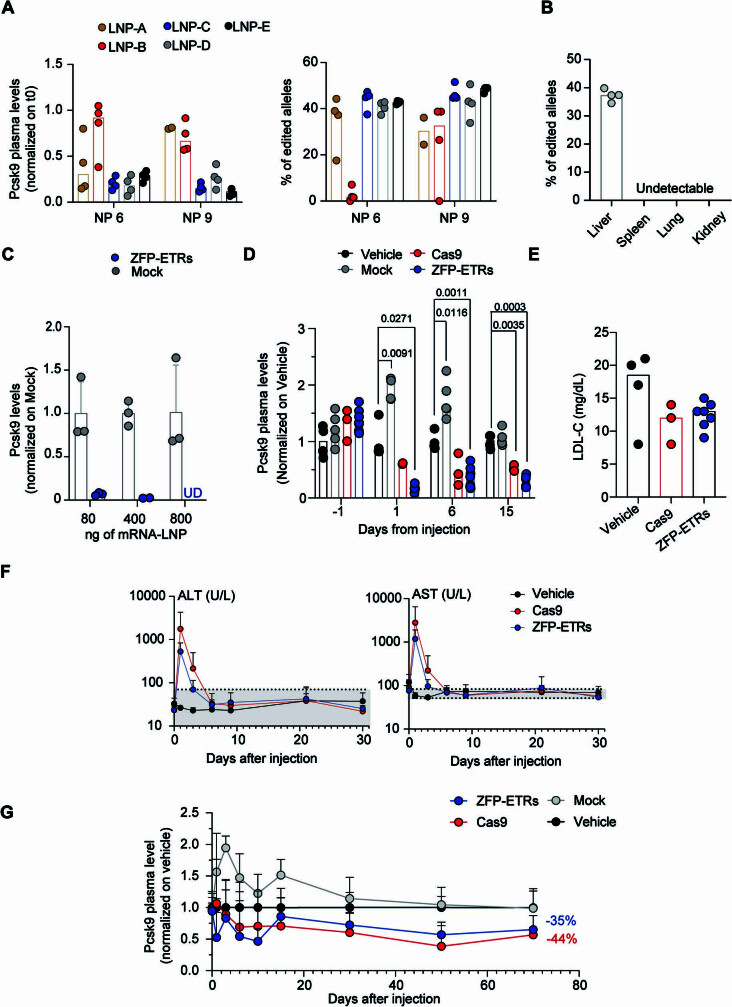
Editing of *Pcsk9* in mice. **a**, Bar plots showing the circulating levels of Pcsk9 (left) and percentage of edited alleles (right) at day 7 post-injection of the indicated LNP formulations encapsulated with Cas9-encoding mRNA and a sgRNA targeting the first exon of *Pcsk9* (n = 4 for each group). Treatments with LNP A (NP ratio 9) and LNP-E (NP ratio 9) resulted in 2 and 1 death, respectively. Dots: data from individual mice normalized to pre-treatment levels. Bars: median for each group. **b**, Bar plot showing the percentage of *Pcsk9* edited alleles from different organs of mice treated with LNP D (NP ratio 6) encapsulated with Cas9-encoding mRNA and a sgRNA targeting the first exon of *Pcsk9*. Dots: data from individual mice (n = 4). Bars: median for each group. **c**, Bar plot showing levels of Pcsk9 in the supernatants of mouse hepatocytes after transfection of three different doses of mRNAs encoding for either ZFP-ETRs or eGFP (mock). Pcsk9 levels for each replicate and each group were normalized to the mean of the mock at the same dose. Data from individual replicates are reported as dots; bars indicate average values (n = 3, mean ± s.d.). UD: undetectable. **d**, Bar plot showing the levels of Pcsk9 in the plasma of mice treated as indicated in Fig. 3a,b of the main text. Data for individual mice (dots) are reported as normalized to the mean of vehicle-treated mice. Bars indicate the median for any conditions. Data are reported as mean ± s.d. (n = 7 for ZFP-ETR-, 3 for Cas9-, 5 for mock- and 4 for vehicle-injected mice).Statistical analysis by two-way RM ANOVA and Dunnett’s multiple comparisons test between vehicle and the other treatment conditions at the lates time point of analysis; *P* values are reported in the figure. If not indicated, differences were not statistically significant. **e**, Bar plots showing the circulating levels of LDL-C in mice 30 days after the indicated treatments (n = 7 for ZFP-ETR-, 3 for Cas9-, 5 for mock- and 4 for vehicle-injected mice). Dots: individual mice. Bars: median level for each group. **f**, Time course of transaminases (ALT and AST) until day 30 post-treatment. Data are reported as the mean ± s.d. of U/L of plasma (n = 6 for any groups). Grey area indicates physiological levels. **g**, Time course of circulating Pcsk9 until day 70 post-treatment. Data are reported as the mean ± s.d. and normalized to PCSK9 levels in vehicle-treated mice (n = 22 for ETR-, 7 for Cas9-, 16 for mock- and 15 for vehicle-injected mice). Source data

In parallel with these studies, we set out to deliver the ZFP-ETRs to the liver of mice. As the ETR technology entails the use of transient gene-delivery modalities, we performed an in vivo screen to identify lipid nanoparticles (LNPs)^28^ compatible with the transfer of editing machinery to the liver. Here, we used CRISPR–Cas9–mediated inactivation of *Pcsk9* as a surrogate readout for LNP-mediated delivery of editors’ RNAs. Yet, in the *Pcsk9* editing field, CRISPR–Cas9 represents a benchmark for emerging editing modalities. Among the ten LNPs tested, seven induced a robust reduction in the circulating levels of PCSK9 (more than 60%) and efficient gene editing (Extended Data Fig. 3a). Of these seven candidates, LNP D was selected for further studies, given its favourable biodistribution and toxicity profiles (Extended Data Fig. 3b). We then produced the mRNAs that encode for the three ZFP-ETRs with state-of-the-art modifications intended to maximize RNA translation and stability and minimize innate immune responses^29^. These mRNAs were packaged into the selected LNP formulation for an initial test in cultured primary mouse hepatocytes, in which we observed a near-complete loss of PCSK9 in the cells’ supernatant (Extended Data Fig. 3c). On the basis of these results, we administered intravenously the ETR-loaded LNPs to adult C57BL/6 mice and monitored circulating levels of PCSK9 for up to 330 days, when the experiment was terminated (Fig. 3a). Mice treated with PBS (hereafter, vehicle) or injected with LNPs loaded with an eGFP mRNA (hereafter, mock) were used as controls. Early analyses of ETR-treated mice showed a rapid and profound reduction in PCSK9, which then stabilized at around 40% of vehicle-treated levels until the last time point analysed (Extended Data Fig. 3d and Fig. 3b). In line with these data, at day 30 after LNP injection, the levels of LDL-associated cholesterol (LDL-C) were reduced in ETR-treated mice (around 35%; Extended Data Fig. 3e). Comparable efficiencies and kinetics of PCSK9 and LDL-C reduction were observed in mice that were treated with LNPs loaded with *Pcsk9*-targeted CRISPR–Cas9 RNAs (Fig. 3b and Extended Data Fig. 3d,e). Treatment-related toxicities were self-contained, with transient increases in the liver enzymes alanine transaminase (ALT) and aspartate aminotransferase (AST) at levels comparable between CRISPR–Cas9- and ZFP-ETR-treated groups (Extended Data Fig. 3f). Because a previous study did not show any significant liver toxicity when using the same *Pcsk9-*targeting CRISPR–Cas9 components but loaded in a different LNP formulation^24^, we concluded that the enzyme increases we observed here were probably a result of the LNP formulation used in this study. To confirm and extend our findings, we treated a second cohort of mice with ETR-loaded LNPs (Extended Data Fig. 3g) and, three months later, subjected four of them to partial hepatectomy (Fig. 3c), a surgical procedure that induces robust waves of hepatocyte proliferation to regenerate the resected liver lobules^30^. Notably, no major differences in the circulating levels of PCSK9 were found between pre- and post-hepatectomized mice, providing further support for the stability of epi-silencing even after active cell replication (Fig. 3d). Similar results were obtained in mice in which *Pcsk9* was genetically inactivated. We also compared the CpG methylation profile of the *Pcsk9* gene promoter in pre-hepatectomized mice treated with ETRs or vehicle and found a net increase in DNA methylation in the former group (Fig. 3e). These levels remained stable also after partial hepatectomy (more than two months), further corroborating the durability of epigenetic silencing through liver regeneration.

## Improved epi-silencing by EvoETRs

**Fig. 4:**
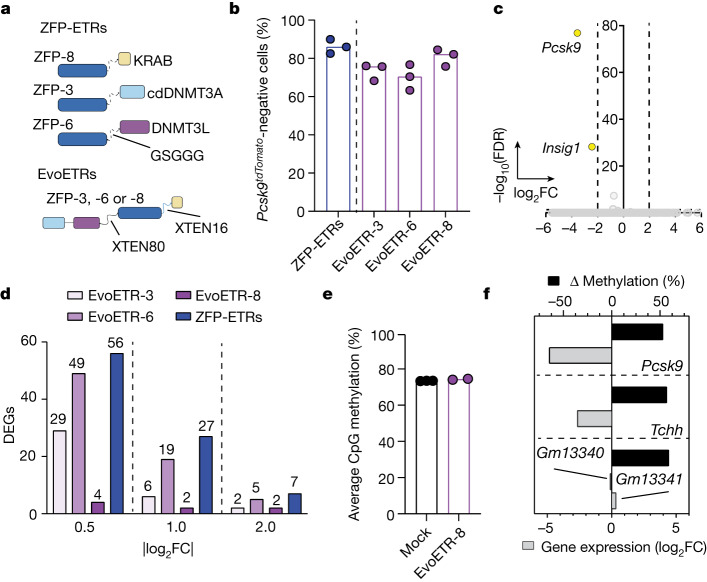
In vitro efficacy and specificity profiling of EvoETRs. **a**, Schematic representation of the molecular architecture of ZFP-ETRs and EvoETRs. Created with BioRender.com. **b**, Bar plot showing the percentage of *Pcsk9*-silenced cells at day 40 after the delivery of either ZFP-ETRs (blue) or EvoETRs (purple). Dots represent individual percentages; bars represent the median for each treatment (*n* = 3). **c**, Volcano plot from RNA-seq analyses showing differential gene expression between EvoETR-8-treated and untreated cells (*n* = 2). The Wald test for binomial distributions was applied for differential gene expression analysis and *P* values were corrected for multiple testing using the Benjamini–Hochberg approach. Dashed lines indicate the thresholds on adjusted *P* values (FDR ≤ 0.05) and fold change (|log_2_FC| ≥ 2). Downregulated genes are in yellow. Genes in grey are not differentially expressed according to the applied thresholds. **d**, Bar plot showing the number of DEGs from the indicated samples versus untreated cells. These analyses were performed at three different |log_2_FC| thresholds and at FDR ≤ 0.05. **e**, Bar plot showing the genome-wide levels of CpG methylation of the indicated samples as calculated from the WGMS analyses (*n* = 3 for mock-treated and *n* = 2 for EvoETR-8-treated cells). **f**, Bar plot showing the correlation between differential methylation and variation in gene expression for the comparison EvoETR-8 versus mock. DMRs were associated with a given gene when falling into a ±10-kb window around its own TSS. Plotted are genes for which the log_2_FC and FDR can be computed from the differential expression analysis. Black bars indicate the variation in the average methylation levels of the DMRs. Source data

**Extended Data Fig. 4:**
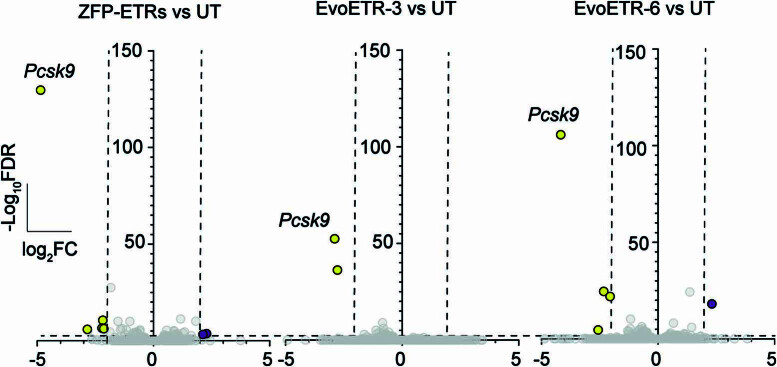
In vitro characterization of the specificity of EvoETRs. Volcano plots from RNA-seq analyses showing differential gene expression between the indicated ETR-treated samples and untreated cells (*n* = 2). Purple and yellow dots indicate genes significantly up- and downregulated, respectively; grey dots indicate genes considered not differentially expressed. Thresholds were set at FDR ≤ 0.05 and |log_2_FC| ≥ 2 and are indicated in the graphs as dashed lines. Source data

Finally, with the aim of reducing the molecular complexity of the epi-silencing platform, the application of which requires three independent mRNAs to be produced and co-delivered, we converted the triple-ETR combination into an all-in-one molecule (a design collectively referred to as evolved ETR; EvoETR). To this end, we appended at the N terminus of the ZFP DBD an obligate heterodimer between cdDNMT3A and DNMT3L and, at the C terminus, the KRAB domain (Fig. 4a), a design reminiscent of previously described epigenetic editors^21,22^. To assess which of the three ZFP arrays used in the triple-ETR combination (ZFP-3, ZFP-6 or ZFP-8) was best-suited for an all-in-one configuration, we built EvoETRs for each of these arrays and assessed their epi-silencing efficiency in the Hepa 1-6 *Pcsk9*^*tdTomato*^ cells. All constructs produced high and durable levels of *Pcsk9* silencing, which were comparable with those obtained with the triple-ETR combination (Fig. 4b). Transcriptional profiling of *Pcsk9*-silenced cells from all treatment conditions showed that the EvoETR equipped with ZFP-8 (hereafter, EvoETR-8) was the most specific one, and this was true across ascending fold-change thresholds (from 0.5 to 2 |log_2_FC|, FDR ≤ 0.05; Fig. 4c,d, Extended Data Fig. 4 and Supplementary Table 3). None of these differentially expressed genes (DEGs; up to four at |log_2_FC| > 0.5) were shared with the triple-ETR combination, in contrast with the EvoETRs equipped with ZFP-6 and ZFP-3, which shared up to ten and three DEGs—including *Pcsk9*—with the triple-ETR combination, respectively (|log_2_FC| ≥ 0.5, FDR ≤ 0.05; Fig. 4d and Supplementary Tables 3 and 4). The genome-wide levels of CpG methylation were largely superimposable between mock- and EvoETR-8-treated cells (Fig. 4e). The latter exhibited five DMRs, three of which annotated to four genes (Fig. 4f and Supplementary Table 5), including *Pcsk9*, the only gene showing significant downregulation (|log_2_FC| ≥ 0.5, FDR ≤ 0.05). These numbers were lower than those observed with the triple-ETR combination (Fig. 2c and Supplementary Table 2). Altogether, these data point to EvoETR-8 as the best-performing and specific reagent for *Pcsk9* silencing. Furthermore, they indicate that the perturbations observed with the triple-ETR combination were at least in part due to the off-targeting of ZFP-3 and ZFP-6, both of which also showed an individual off-targeting profile.

**Fig. 5:**
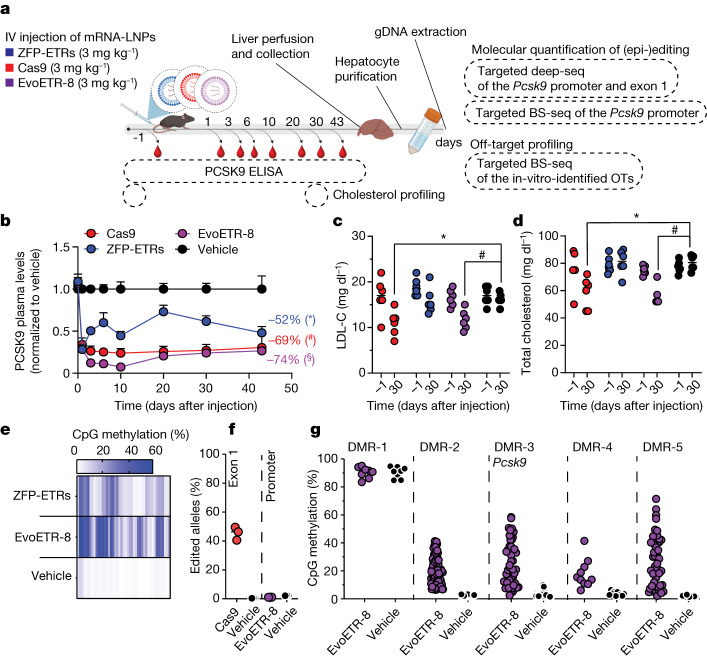
Improved epi-silencing of *Pcsk9* in vivo after LNP-mediated delivery of EvoETR-8. **a**, Schematic drawing of the experimental procedures. LNPs were loaded with mRNAs encoding ZFP-ETRs, Cas9 or EvoETR-8 and separately injected intravenously into mice. Vehicle, PBS-treated mice. Before and after LNP injection, blood samples were collected to measure the circulating levels of PCSK9 and cholesterol. Genomic DNA (gDNA) from purified hepatocytes at day 43 after injection was analysed to measure the efficiency of genetic or epigenetic editing at *Pcsk9* by targeted deep sequencing (deep-seq) or BS-seq, respectively. DNA methylation levels were also quantified by targeted BS-seq of in-vitro-identified DMRs. OTs: off targets. Created with BioRender.com. **b**, Time course of the levels of circulating PCSK9 up to 43 days aftr LNP injection. Data are mean ± s.d. (*n* = 6). Statistical analysis by two-way RM ANOVA and Dunnett’s multiple comparisons test between vehicle and the other treatment conditions at the latest time point (*P = 0.0451, #*P* = 0.0117 and §*P* = 0.0118). If not indicated, differences were not statistically significant. **c**,**d**, Dot plots showing the levels of LDL-C (**c**) and total cholesterol (**d**) in mice 30 days after the treatments (*n* = 6). Dots represent individual mice; lines represent the median for each group. Statistical analysis by two-way RM ANOVA and Dunnett’s multiple comparisons test (*P = 0.0025 and #*P* = 0.0090 for LDL-C; *P = 0.0001 and #*P* < 0.0001 for total cholesterol). If not indicated, differences were not statistically significant. **e**, Heat map showing the average methylation of single CpGs within the *Pcsk9* CGI of treated (ZFP-ETRs and EvoETR-8) and control (vehicle) mice. Colour intensity refers to the percentage of methylation (*n* = 3). Each rectangle represents an individual CpG in the genomic region Chr. 4: 106,463,706–106,464,363. **f**, Dot plot showing the percentage of edited alleles. Data are reported as percentages of individual mice (dots) and medians (lines). The percentage of edited alleles was measured in *Pcsk9* exon 1 for Cas9- and vehicle-treated mice, and in the *Pcsk9* promoter for EvoETR-8- and vehicle-treated mice (*n* = 3). **g**, Dot plots showing the percentage of in vivo methylation in EvoETR-8- and vehicle-treated mice by targeted BS-seq. Five genomic sites were interrogated, corresponding to the five DMRs that were identified in vitro from the comparison EvoETR-8 versus mock. Each dot represents a single CpG in the indicated DMR (mean of *n* = 3). The plot showing the *Pcsk9*-associated DMR (DMR-3) is a reanalysis of the data in **e** and was included here as reference for on-target methylation. Source data

**Extended Data Fig. 5:**
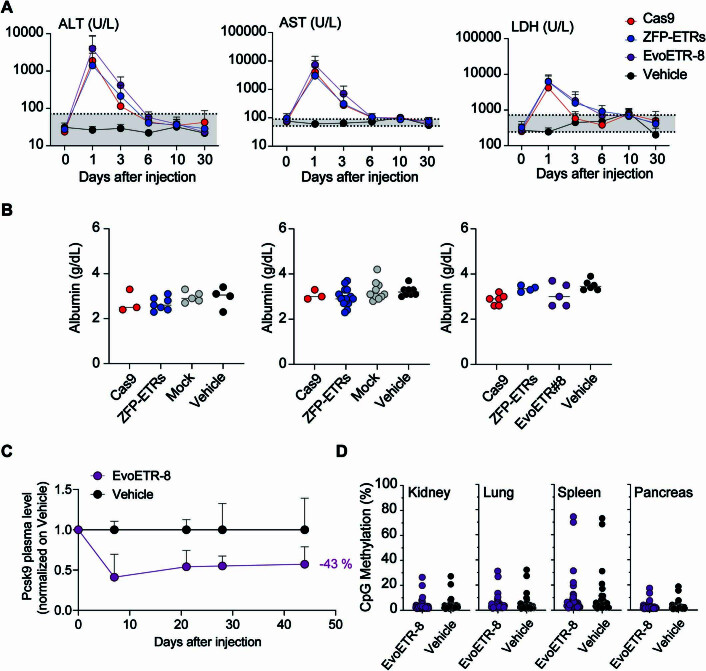
Efficacy, biodistribution and liver toxicity of LNP-mediated delivery of ETRs. **a**, Time-course analysis of plasma levels of ALT, AST and LDH after LNP-mediated delivery of mRNAs encoding for either Cas9, ZFP-ETRs, or EvoETR-8. Data are reported as mean ± s.d. (n = 6). **b**, Quantification of circulating albumin at the last time point analysed. Data are reported as individual values (dots) and medians (lines). Left: experiment in Fig. 3b, 330 days post-injection; centre: experiment in Extended Data Fig. 3g, 70 days post-injection; right: experiment in Fig. 5b, 43 days post-injection. **c**, Time course of circulating Pcsk9 until day 44 post-injection of GenVoy-LNPs encapsulating the mRNA of EvoETR-8. Data are reported as mean ± s.d. and normalized to the PCSK9 levels of vehicle-treated mice (n = 5 for EvoETR-8- and 2 for vehicle-injected mice). **d**, Dot plots showing the percentage of CpG methylation at the *Pcsk9* promoter in the indicated organs from EvoETR-8- and vehicle-treated mice. Each dot represents a single CpG (mean of n = 3). Source data

**Extended Data Fig. 6:**
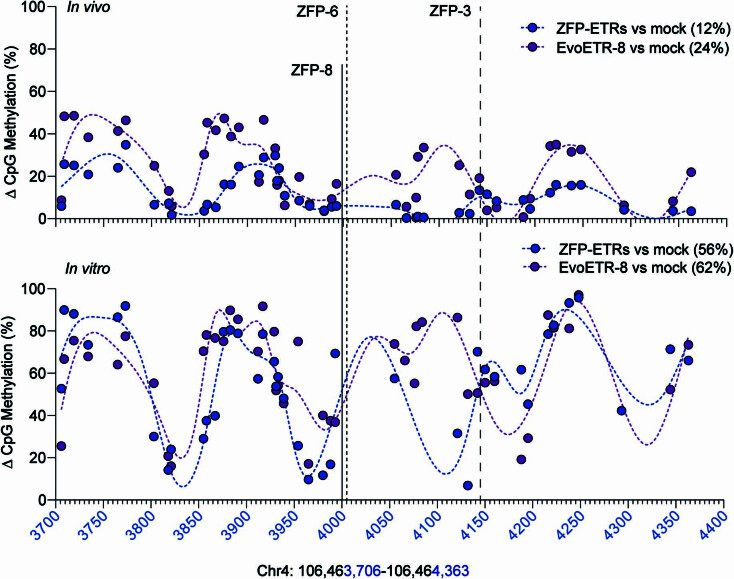
CpG methylation profiles at *Pcsk9* in vivo and in vitro. Dot plots reporting the delta methylation between either ZFP-ETR- or EvoETR-8-treated samples versus untreated controls. Individual dots indicate the average delta methylation of each CpG in the genomic region Chr.4: 106,463,706–106,464,363. Connecting lines were defined as smoothing spline with 20 knots. Mean delta methylation throughout the entire windows for each sample are reported in brackets. Top: replotting of the targeted BS-seq data of Fig. 5e. Bottom: replotting of the WGMS analysis of Fig. 4e,f (for EvoETR-8) and Fig. 2d for ZFP-ETRs. Positions of ZFP-binding sites are indicated as continuous (ZFP-8) or dashed (ZFP-6 and ZFP-3) lines. Source data

**Extended Data Fig. 7:**
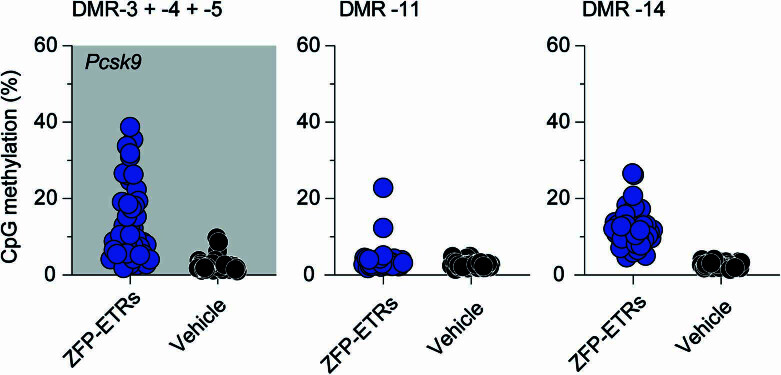
In vivo methylation profile of the top three DMRs identified in vitro from the ZFP-ETRs versus mock comparison. Dot plots reporting the percentage of in vivo methylation at single-CpG resolution in ZFP-ETR- and vehicle-treated mice by targeted BS-seq. Two genomic sites were interrogated corresponding to the top two DMRs identified in vitro from the ZFP-ETRs versus mock comparison. DMR-3, -4 and -5 were identified in vitro as associated with the *Pcsk9* promoter region. The in vivo methylation levels of these DMRs were quantified producing a single PCR including all the three DMRs. Each dot represents a single CpG in the indicated DMRs (mean of n = 3 for each experimental group). Source data

We then delivered the mRNA of EvoETR-8 into mice by LNPs and observed a reduction of around 75% in the circulating levels of PCSK9 until day 43 (Fig. 5a,b). Similar results were obtained after the injection of matched amounts of LNPs loaded with the *Pcsk9-*targeting CRISPR–Cas9 components (around 70%), whereas epi-silencing with the triple-ETR combination led to a 50% reduction in the levels of circulating PCSK9 (Fig. 5a,b). ALT, AST and lactate dehydrogenase (LDH) were transiently increased in all LNP-treated groups (Extended Data Fig. 5a), whereas LDL-C and total cholesterol were significantly reduced in both EvoETR-8-treated mice (29 and 26%, respectively) and gene-edited mice (34 and 22%, respectively; Fig. 5c,d). The levels of liver-secreted albumin were comparable among treatments (Extended Data Fig. 5b), indicating that targeted activity of the different editors—rather than treatment-related effects—was the basis of the observed reductions in PCSK9 across all in vivo experiments. For EvoETR-8, we also tested another LNP, and still obtained a significant inhibition of *Pcsk9*, confirming that the epi-silencing platform is transferrable to other particle formulations (Extended Data Fig. 5c). EvoETR-8 was also more efficient than the triple-ETR combination in terms of deposing DNA methylation at the *Pcsk9* promoter (1.9-fold increase in mean methylation rate; Fig. 5e and Extended Data Fig. 6). This increase was associated with increased methylation at CpG sites that were either poorly or not methylated in triple-ETR-treated mice. Of note, the CpG methylation profiles at *Pcsk9* after treatment with ZFP-ETRs or EvoETR-8 were superimposable between the in vivo and the in vitro conditions (Extended Data Fig. 6). No differences in the levels of CpG methylation at the *Pcsk9* promoter were detected in the lung, spleen, kidney or pancreas of ETR-treated mice versus mock-treated mice (Extended Data Fig. 5d), further confirming the targeting specificity of the LNPs. Additional molecular analyses showed no signs of genetic alterations at the *Pcsk9* promoter of mice treated with EvoETR-8, whereas the mutational burden imposed by CRISPR–Cas9 at its intended target site reached 45% (Fig. 5f). Finally, we inspected the four unintended DMRs identified in vitro in purified hepatocytes from EvoETR-8-treated mice and confirmed three of them; for the remaining one, the interrogated CpGs were already highly methylated in mock-treated mice (Fig. 5g). In line with these data, the top three DMRs identified in vitro from the triple-ETR combination were hypermethylated in the corresponding treated mice (Extended Data Fig. 7). These data confirm the off-target nature of these sites and support the predictive value of the in vitro studies for off-target nomination.

## Discussion

In this study, we show that LNP-mediated delivery of mRNAs encoding ETRs to the liver of mice can lead to durable (nearly one year of follow-up) epigenetic silencing of *Pcsk9*. Notably, epi-silencing proved to be stable also after partial hepatectomy, further confirming the heritable nature of the epigenetic marks deposited by the ETR technology and indicating that epi-silenced hepatocytes remained competent for liver regeneration. When compared with RNAi, for which multiple administrations are required^31^, our approach is configured as a one-and-done treatment, a feature shared only with other genome-editing technologies. Unlike the latter approaches, however, the ETR technology does not require the induction of potentially genotoxic DNA breaks to inactivate the desired gene^32–34^. This feature represents a safety advantage, especially when aiming at multiplex epi-silencing, as both gene editing, and to a lesser extent, base editing can cause reciprocal chromosomal translocations^33,35,36^. Moreover, epi-silencing differs from genome editing in that it can be reverted by either pharmacological intervention or treatment with editors equipped with a transcriptional activator, as previously shown in cell lines^15,22^. As such, epi-silencing would allow for temporally controlled silencing of the targeted gene and the reversal of treatment-related adverse effects. Here, we also show that the ETR technology can establish substantial levels of epi-silencing in vivo, at values that are already compatible with several experimental and therapeutic applications. In our experimental settings, epi-silencing performed as well as conventional gene editing (up to 75% of *Pcsk9* inhibition). Of note, the same CRISPR–Cas9 components used in this study were shown to completely abrogate *Pcsk9* expression when delivered by another LNP in mice^24^, which suggests that using more efficient and tolerated non-viral delivery platforms could further increase epi-silencing efficiency. In addition, one might follow the same molecular optimization strategies used for base editors^29,37,38^ that, in their latest versions, promote near-complete abrogation of *Pcsk9* expression in both mice and non-human primates^25^. For the epi-silencing technology, these optimizations could include further refinements in the RNA payload and/or the ETR design, the identification of better-responding ETR target sites from a larger repertoire and the use of other types of DBD. Indeed, our initial molecular optimization of ETR architecture already improved the epi-silencing efficiency in vivo. The stronger performance of EvoETR-8 as compared with the parental ETR combination could be attributed to intrinsic characteristics of the all-in-one fusion construct, differences in the LNP packaging efficiencies and/or the mRNA structure or stability. With regard to the in vivo durability of *Pcsk9* repression with EvoETR-8, this proved to be stable until day 43, the last time point analysed. It is conceivable that mice treated with EvoETR-8 will maintain the many-month-long silencing observed with the triple-ETR combination, given that these two platforms share the same epigenetic effector domains and that the levels of on-target DNA methylation were even higher in EvoETR-8-treated mice. Because the resulting increase in on-target activity obtained from some of these optimizations might also result in higher levels of off-targeting, these steps should be paralleled by a thorough specificity assessment through combinations of transcriptional and genome-wide epigenetic analyses. In this regard, we found here that treatment with the ZFP-based ETRs results in limited transcriptional and epigenetic perturbations. Whereas the exact mechanisms behind this phenomenon are still unknown, multiple lines of evidence point to unintended docking of the ZFP arrays at off-target sites rather than in-solution activity of the effector domains. Indeed, we and others did not detect any perturbations with untargeted ETRs or dCas9-based epi-editors^15,22^. Moreover, single-molecule ETRs equipped with alternative ZFP arrays induce diverse numbers and types of differentially expressed genes. Of note, testing of three different ZFP arrays identified a highly specific one, suggesting that empiric selection of DBDs from a larger catalogue might enable the identification of ETRs with undetectable off-target activity. In vivo confirmation of in-vitro-nominated off-target sites might indicate that ETR activity is invariant in these two biological systems, a hypothesis that needs further confirmation with comprehensive genome-wide epigenetic and transcriptomic analyses. In line with previous reports, our in vitro data show that the ETR technology is applicable to different DBD platforms^15,21,22^, with ZFP-based ETRs showing a more favourable efficiency profile than do dCas9- and TALE-based architectures. Although this finding requires further confirmation (for example, through a systematic evaluation of a larger panel of DBDs and genomic loci), intrinsic features of the ZFP platform make it appealing for epigenome editing applications. These include their reduced molecular size and independence from short-lived gRNAs for activity, characteristics that would facilitate both the delivery and the stability of the epigenome editing complex. Alternatively, we can speculate that ZFP-based ETRs could be more proficient than are dCas9- or TALE-based architectures in tethering or placing the epigenetic EDs onto chromatin, given their structural similarities with naturally occurring ZFP-based transcriptional repressors. In conclusion, we establish here a proof-of-principle of durable and efficient epigenetic silencing in vivo by transient ETR delivery, opening exciting possibilities in the field of gene therapy.

## Methods

### Molecular cloning and mRNA production

ETRs were transiently delivered as either plasmid DNA (in cell lines) or mRNA (in cell lines, primary cells and in vivo). To this end, ETRs were cloned in an expression vector containing: (i) an upstream CMV promoter; (ii) an upstream T7 promoter for mRNA in vitro transcription (IVT); (iii) a downstream WPRE signal; (iv) a 3′-terminal stretch of 64 adenines (64A); and (v) a SpeI plasmid linearization site for mRNA IVT (Extended Data Fig. 1b). The coding sequences of the dCas9-based ETRs were previously described^15^. For plasmid-mediated gRNA expression, the crRNA sequences were cloned into a previously described expression vector containing a U6 promoter and the sequence of the *Staphylococcus pyogenes* Cas9 trRNA^39^. gRNAs targeting the CGI of the mouse *Pcsk9* were designed using Chop Chop^40^ (https://chopchop.cbu.uib.no/) and selected according to high simulated activity and specificity. ZFPs and TALEs were designed and synthesized by Merck and Thermo Fisher Scientific, respectively, and subcloned into the mammalian expression plasmid in place of the dCas9 sequence. mRNAs were produced by IVT using the T7 Megascript Kit (Thermo Fisher Scientific, AMB1334-5) according to the manufacturer’s instructions. For the in vitro experiments, partially modified mRNAs were produced by IVT, including the following modifications to the standard protocol: (i) inclusion of the anti-reverse cap analogue 3´-O-Me-m7G(5′)ppp(5′)G (NEB, M0251) at a final concentration of 8 mM; and (ii) reduction of the GTP concentration from 7.5 to 2.5 mM. For the in vivo experiments, heavily modified mRNAs were produced by IVT, including the following modifications to the standard protocol: (i) inclusion of CleanCap-AG (Trilink BioTechnologies, N-7113) at a final concentration of 4 mM; and (ii) substitution of UTP with N1-Met-ψ-Uridine (Trilink BioTechnologies, N-1081) at a final concentration of 7.5 mM. mRNAs were then purified using the RNeasy Mini Kit (Qiagen, 74134). The quality and integrity of the mRNAs were assessed with a 4200 TapeStation System, and quantities were measured by a NanoDrop 8000. sgRNAs were synthetized by Axolab according to the a previously described nucleotide-modification scheme^41^. For in vitro or in vivo studies, the Cas9 mRNA was in vitro transcribed as described above or purchased from Trilink BioTechnologies (L-7606), respectively. Sequences of the ETRs and gRNAs used in this study are listed in Supplementary Table 6. The plasmids used in this study are available upon signing of a material transfer agreement.

### Cell culture, treatment and engineering

Hepa 1-6 cells (CRL-1830, ATCC) were cultured in Dulbecco’s modified Eagle’s medium (DMEM, Corning, 10-013-CV) supplemented with 10% fetal bovine serum (FBS; EuroClone), 1% l-glutamine (EuroClone, ECB3000D) and 1% penicillin–streptomycin (Euroclone, ECB3001D) at 37 °C in a 5% CO_2_ humidified incubator. The Hepa 1-6 *Pcsk9*^*tdTomato*^ cell line was generated by nucleofecting 3 × 10^5^ Hepa 1-6 cells with: (i) an HDR donor plasmid containing the 2A-tdTomato-polyA cassette within homology arms to exon 12 of *Pcsk9*; (ii) a Cas9-expression plasmid; and (iii) a plasmid expressing a gRNA targeting the last exon of *Pcsk9* (ref. ^42^). tdTomato-positive cells were than sorted at single-cell level and amplified. The Hepa 1-6 *Pcsk9*^*tdTomato*^ cell line is available upon signing of a material transfer agreement. Primary mouse hepatocytes from C57BL/6 male mice were purchased from Biopredic International as adherent monolayers on collagen-coated 96-well plates and maintained according to the manufacturer’s instructions. Supernatants of treated and control cells were collected at different time points and stored as one-time-use aliquots at −20 °C.

### Gene-delivery procedures

For the in vitro experiments in the Hepa 1-6 *Pcsk9*^*tdTomato*^ cells, 3 × 10^5^ cells were transfected with either RNAs or plasmid DNAs using the 4D-Nucleofector X System (Lonza) in SF Cell Line solution (Lonza, V4XC-2032) and with the CM-137 pulse program. For the in vitro and in vivo experiments with LNPs A, B, C, D and E, these research-grade reagents were formulated by Precision NanoSystem (PNI) combining lipid mixes and RNA, the latter dissolved in a PNI proprietary formulation buffer. The lipid mixes are made of four different components dissolved in ethanol-based solution: an ionizable lipid, a helper lipid, cholesterol and 1,2-dimyristoyl-rac-glycero-3-methoxypolyethylene glycol (PEG-DMG). The chemical nature of the helper lipid differs among the formulations: (i) 1,2-di-(9Z-octadecenoyl)-*sn*-glycero-3-phosphoethanolamine (DOPE) for LNP A; (ii) 1,2-dioctadecanoyl-*sn*-glycero-3-phosphoethanolamine (DSPC) for LNP B; (iii) 1,2-dipalmitoyl-*sn*-glycero-3-phosphocholine (DPPC) for LNP C; and (iv) 1,2-di-(9Z-octadecenoyl)-*sn*-glycero-3-phosphocholin (DOPC) for LNPs D and E. The ionizable lipid as well as the molar ratio at which the four components were mixed are proprietary information of PNI. RNAs and lipids were mixed into the NanoAssemblr Ignite instrument (PNI) using microfluidic cartridges (Ignite NxGen Cartridge; PNI) with a total flow rate (TFR) of 12 ml min^−1^ and a flow rate ratio (FRR) of 3:1 (RNAs:lipids). Nitrogen-to-phosphate (NP) ratios of 6 and 9 were used in the initial LNP screening experiments, and this ratio was set to 6 for the remaining in vivo experiments. The lipid mix LNP D is available upon request from Precision NanoSystems (PNI) using the code iL00V77. LNP D formulated with the desired RNA can be directly purchased from PNI by signing an agreement. The estimated turnaround time is 1–2 months. Alternatively, the LNP D and the formulation device can be purchased from PNI. In this case, PNI will technically support the investigator in the setting of the formulation protocol. LNPs produced with the GenVoy-ILM reagent (NWW0042, 25 mM) were formulated following the manufacturer’s instructions with the NanoAssemblr Ignite instrument (PNI) and using microfluidic cartridges (Ignite NxGen Cartridge; PNI). Formulation parameters were set as follows: (i) TFR of 12 ml min^−1^; (ii) FRR of 3:1; and (iii) NP ratio of 4. All LNPs were concentrated using an Amicon Centrifugal Filter (MWCO 30 kDa), the ethanol was removed by a 3:1 dilution in 1× PBS (pH7-7.3, Mg2^+^/Ca2^+^- free) and LNPs were finally filtered manually through a 0.22-µm syringe. Particle sizes and their polydispersity index (PDI) were analysed using dynamic light scattering (DLS), and the RNA encapsulation efficiency and concentrations were determined using a RiboGreen plate-based assay. The results of the DLS analyses and RNA quantification of the LNPs used in this study are reported in Supplementary Table 7.

### Flow cytometry and cell sorting

Flow cytometry was performed using CytoFLEX S (Beckman Coulter) and raw data were analysed using FCS Express v.7 (DeNovo Software) to extract the percentage of *Pcsk9*^*tdTomato*-^negative cells. When indicated, tdTomato-positive or -negative cells were sorted with a FACSAria Fusion Cell Sorter (BD Biosciences) as either bulk populations or at the single-cell level. The gating strategy for both the flow cytometry and the cell sorting procedures is reported in Supplementary Fig. 1.

### RNA sequencing

Total RNA was extracted from 6 × 10^6^ *Pcsk9*-silenced cells using the RNeasy Mini Kit (Qiagen, 74134) and quantified using the Qubit 2.0 Fluorimetric Assay (Thermo Fisher Scientific). Unstranded libraries were prepared with the NEBNext Ultra RNA Library Prep Kit for Illumina after rRNA depletion, and sequencing was performed using an Illumina NovaSeq 6000 platform (NovaSeq Control Software v.1.7) to obtain at least 30 million of 150bp-long paired-end reads per sample. Read quality was controlled with Fastqc v.0.11.9 (https://www.bioinformatics.babraham.ac.uk/projects/fastqc/), and low-quality reads and the adapters were removed using Trim Galore v.0.6.6 (https://www.bioinformatics.babraham.ac.uk/projects/trim_galore/) according to the following parameters: --quality 20, --length 25, --paired. High-quality remaining reads were aligned to the mouse reference genome GRCm38 using STAR v2.7.6a (ref. ^43^) with default parameters. Gene counts were quantified using the featureCounts function from the Subread package v.2.0.1 (ref. ^44^) and Gencode M25 as the gene model. Raw counts were corrected for biases due to different library preparations, if present, using the ComBat_seq function from the R Bioconductor package sva v.3.38.0 (ref. ^45^). Read distribution was estimated using the negative binomial generalized log-linear model implemented in the R Bioconductor package DESeq2 v.1.30.0 (ref. ^46^). Differential gene expression was tested using the nbinomWaldTest function and *P* values were corrected using the Benjamini–Hochberg approach.

### WGMS

Genomic DNA was extracted from 6 × 10^6^ *Pcsk9*^*tdTomato*^-silenced cells using the Maxwell RSC Cultured Cell extraction kit (AS1620) and quantified using a NanoDrop 8000. Libraries were prepared using an enzymatic approach for cytosine conversion with NEBNext DNA Ultra II Reagents and sequencing was performed using an Illumina HiSeq (HiSeq Control Software v.3.4) platform to produce at least 250 million 150-bp-long paired-end reads per sample. Read quality was controlled with Fastqc v.0.11.9, and low-quality reads and the adapters were removed using Trim Galore v0.6.6. with the following parameters: --quality 20, --length 25, --paired, --clip_R2 5. High-quality remaining reads were analysed using the Bismark read mapper methylation caller tool v.0.23.0. In detail, reads were aligned to both the converted and the unconverted genomes (GRCm38) using Bismark v.0.23.0 with default parameters. Duplicates were then removed using the deduplicate_bismark script and the methylation status was obtained using the bismark_methylation_extractor script. Then, the methylation call was loaded into the R environment and processed using the R Bioconductor package MethylKit v.1.16.1. Imported data were filtered using the filterByCoverage function (low count filter equal to 1 and high percentile equal to 99.9) and normalized using the normalizeCoverage function. Information from the different samples was merged using the unite function considering the positions covered in all replicates. The percentage of methylation was calculated with the percMethylation function and the correlation among the samples was determined applying the cor function (default Pearson method). Differential methylation analysis was performed using the R Bioconductor packages bsseq v.1.26.0 (ref. ^47^) and DSS v.2.44.0 (ref. ^48^). First, the object was created using the makeBSseqData function starting from the Bismark output. The DMLtest function was used for the normalization step and the differential analysis with the following parameters: smoothing = TRUE and smoothing.span = 500. Then, the callDML function was applied to determine the differential methylated loci (DML) setting as thresholds delta = 0.4 and p.threshold = 1 × 10^−3^. To exclude any confounding DMRs not associated with off-target methylation, the delta methylation threshold was set at 0.4; that is, the minimal value not calling any DMR in Cas9-treated cells, a negative control having no direct methylation activity. The DMRs were defined applying the callDRM function with the same thresholds. The DMRs identified were annotated using the annotatePeakInBatch function from the R Bioconductor package ChIPpeakAnno v.3.24.2 (ref. ^49^) using the Gencode M25 annotation and the following parameters: PeakLocForDistance = “middle”, FeatureLocForDistance = “TSS”, output = “both” and multiple = TRUE. DMRs of all treated samples were computed using as reference the same mock-treated controls.

### Mouse handling and treatments

Eight-week-old C57BL/6N female mice were purchased from Charles River Laboratories. Procedures involving animal handling and care followed national and international law and policies and were approved by the Institutional Animal Care and Use Committee (authorization numbers 604/2020-PR and 233/2022-PR, provided by the Italian Ministry of Health). Housing temperature and relative humidity were 22 °C (±2 °C) and 55% (±5%), respectively. A 12-h light–12-h light cycle was used and all possible efforts were made to minimize the number of mice used and their suffering. For in vivo administration of either Cas9 or ETRs, mRNA-LNP solutions were diluted in PBS without calcium and magnesium (Corning, 21-031-CV). Subsequently, mice were randomly assigned to a treatment group and heated with an infrared lamp to obtain vasodilatation. Finally, 250 µl of LNP solution or PBS (herein defined as vehicle) were intravenously injected into the tail vein. For plasma analyses (see next section), around 200 µl of blood was collected from the retro-orbital plexus of each experimental mouse by using a non-heparinized micro-haematocrit capillary tube (Kimble Chase, CSX40A502), and then moved into an EDTA-sprayed blood collection tube (Sarstedt, 20.1288.100). Blood was then centrifuged for 10 min at 2,000*g* at room temperature. Purified plasma was finally collected from the supernatant and stored as one-time-use aliquots at −20 °C. For experiment termination and organ collection, mice were euthanized by CO_2_ inhalation and tissues (liver, spleen, lungs and kidney) were removed and snap-frozen for further molecular analyses. For partial hepatectomy, mice were anaesthetized by 2% isoflurane continuous inhalation. Before hepatectomy, mice were fasted for 4 h. Surgery was performed according to the Higgins protocol^50^. In brief, the abdominal skin was shaved, and a 2-cm upper midline incision was made beginning from the xyphoid. After opening the peritoneum, the liver was gently mobilized and exposed. The left lateral lobe was lifted, tied up and resected through 3.0 silk sutures (Ethicon, EH6823H) distal to the applied ligatures. Muscle and skin were closed in two layers with 4.0 Vicryl (Ethicon, V994H) and an autoclip wound-closing system, respectively. For postoperative analgesia, carprofene (5 mg per kg) was used by subcutaneous injection into the neck fat pad. Liver tissue and blood were collected during hepatectomy and at necropsy for molecular analysis and *Pcsk9* plasma quantification. For isolation of hepatocytes, mice were first anaesthetized with isoflurane and the liver was exposed and perfused (32 ml per min) through the inferior vena cava with HBSS-HEPES 0.03% collagenase IV (Sigma). The digested mouse liver was collected, passed through a 100-μm cell strainer (BD Biosciences) and processed into a single-cell suspension. Cells were spun down and washed three times with successive centrifugations at different speeds (30*g*, 25*g* and 20*g*) for 3 min each at room temperature to obtain hepatocytes.

### Plasma analysis

To quantify PCSK9, plasma from treated mice and supernatants from primary mouse hepatocytes were thawed and diluted 1:200 and 1:2, respectively. Dilutions were then loaded on a commercial pre-spotted ELISA kit according to the manufacturer’s instructions (R&D Systems, MPC900). Similarly, absorbance assays were used to quantify the levels of LDL-C (P/N 00018256040, Werfen), ALT (P/N 00018257440, Werfen), AST (P/N 00018257540, Werfen), LDH (P/N 00018258240, Werfen) and albumin (P/N 00 18250040), following the manufacturer’s instructions.

### In vivo molecular analyses

Genomic DNA was extracted from snap-frozen tissues (around 30 mg) using the Maxwell 48 Promega RSC Tissue DNA Purification Kit (AS1610) according to the manufacturer’s instructions. Where indicated, editing and epi-editing efficiencies were quantified from purified hepatocytes (see ‘Mouse handling and treatments’). In these cases, genomic DNA was extracted using the Maxwell 48 RSC Tissue DNA Purification Kit (AS1610) from 1 × 10^6^ cells according to the manufacturer’s instructions. Gene-editing efficiencies at the *Pcsk9* locus were quantified using the T7 assay or targeted deep sequencing. For the T7 assay, a 765-bp genomic region encompassing the CRISPR–Cas9 binding site was PCR-amplified using the primers listed in Supplementary Table 8. PCRs were then processed using the Alt-R Genome Editing Detection Kit (IDT, 1075932), run on the Agilent ScreenTape System, and the percentage of editing was quantified according to the manufacturer’s instructions. For targeted deep sequencing, the promoter region or exon 1 of *Pcsk9* were PCR-amplified using the primers listed in Supplementary Table 8. Libraries were then prepared using the NEBNext Ultra II DNA Library Prep Kit for Illumina for the *Pcsk9* promoter or using the NEBNext Ultra DNA Library Prep Kit for Illumina for *Pcsk9* exon 1, and sequenced using the Illumina MiSeq platform (MiSeq Control Software v.2.6). Sequencing data were analysed with CRISPResso2 v.2.8 (ref. ^51^) to detect nucleotide insertions and/or deletions. Reads were aligned to the boundary sequence around the putative cutting site (400 bp centred on the sgRNA complementary site for Cas9-treated samples or 300 bp centred on the ZFP-8 recognition site for EvoETR-8-treated samples) using bowtie2 v.2.2.5 (refs. ^52,53^) in paired mode and default parameters. After that, original fastq files were subset to retain only the reads mapping to the region of interest using the filterbyname module of the BBMap aligner v.39.01 contained in the BBTools suite (https://sourceforge.net/projects/bbmap/). The remaining reads were analysed with CRISPResso2 in paired-end mode setting the options for Trimmomatic software v.0.39 (ref. ^54^; http://www.usadellab.org/cms/?page=trimmomatic) to remove low-quality positions (score < 30) and Illumina adapters (--trim_sequences --trimmomatic_command trimmomatic --trimmomatic_options_string ‘ILLUMINACLIP:TruSeq3-PE-2.fa:2:30:10 MINLEN:100’). Then, each couple of paired-end reads was merged using FLASH v.1.2.11 (ref. ^55^) to produce a single contig, which was mapped to the input amplicon reference (promoter region or first exon, depending on the experiment). The sgRNA complementary site and the ZFP-8 target sequence were provided to focus the analysis on the target region, and the quantification window was set to 20 bp per side around the cut site (Cas9 samples) or the ZFP-8 middle point (EvoETR-8 samples). Identified alleles were quantified by measuring the number of reads and their relative abundance on the basis of total read counts considering only insertions and deletions. The percentage of CpG methylation at the *Pcsk9* promoter or at the in-vitro-identified DMRs was quantified using targeted bisulfite deep sequencing (targeted BS-seq). Specifically, purified genomic DNA was converted with the EpiTect Fast Bisulfite Conversion Kit (Qiagen, 59104) according to the manufacturer’s instructions. Then, the promoter region of *Pcsk9* and unintended DMRs were PCR-amplified using the primers listed in Supplementary Table 8. For the *Pcsk9* promoter, libraries were prepared using the NEBNext Ultra DNA Library Prep Kit for Illumina. For the other DMRs, the NEBNext Ultra II DNA Library Prep Kit for Illumina was used. Libraries were sequenced using an Illumina MiSeq platform in paired-end mode (MiSeq Control Software v.2.6). Read quality was controlled with Fastqc v.0.11.9, and low-quality reads and adapters were removed using Trim_Galore v0.6.6. with the following parameters: --quality 20, --length 25, --paired, --rrbs. High-quality remaining reads were analysed using the Bismark read mapper methylation caller tool v.0.23.0 (ref. ^56^). In detail, reads were aligned to both unconverted and converted genomes (GRCm38) using Bismark with the --local parameter and the methylation status was obtained using bismark_methylation_extractor script. The methylation calls were loaded into the R environment and processed using the R Bioconductor package MethylKit v.1.16.1 (ref. ^57^). Imported data were filtered using the filterByCoverage function (low count filter equal to 10 and high percentile equal to 99.9) and normalized using the normalizeCoverage function. The data from the different samples were merged using the unite function considering the positions covered in at least one replicate per condition. The percentage of methylation at each CpG was calculated using the percMethylation function.

### Statistics and reproducibility

Data were plotted and analysed using GraphPad Prism v.9 (GraphPad Software). When indicated in the figure legends, statistical significance was evaluated by using GraphPad Prism v.9 (GraphPad Software) and applying the described tests. All of the in vitro experiments were conducted with technical replicates (*n* ≥ 2) and the exact number of replicates is indicated in the respective legend. In vivo experiments were designed including multiple mice (*n* ≥ 3). The exact number of treated mice in any experimental group for any experiment is indicated in the figure legend.

### Reporting summary

Further information on research design is available in the Nature Portfolio Reporting Summary linked to this article.

## Online content

Any methods, additional references, Nature Portfolio reporting summaries, source data, extended data, supplementary information, acknowledgements, peer review information; details of author contributions and competing interests; and statements of data and code availability are available at 10.1038/s41586-024-07087-8.

### Supplementary information

Supplementary Figure 1 Gating strategy for quantification and isolation of Pcsk9^tdTomato^-negative Hepa 1-6 by flow cytometry

Reporting Summary

Supplementary Table 1 Genes differentially expressed in the indicated comparisons (|log_2_FC|≥2, FDR≤0.05).

Supplementary Table 2 Association between Differentially Methylated Regions (DMRs) and expression of closer genes (±10 kb around their TSS)

Supplementary Table 3 Lists of differentially expressed genes from the comparisons between Mock- and either ZFP-ETRs-, EvoETR#3-, EvoETR#6-, or EvoETR#8-treated cells

Supplementary Table 4 Number of differentially expressed genes shared between the ZFP-ETR-treated samples and the indicated EvoETR-treated samples

Supplementary Table 5 Lists of differentially methylated regions (DMRs) and expression of the associated genes for the comparison between EvoETR#8 and Mock

Supplementary Table 6 Amino acidic and nucleotide sequences of the ETRs and nucleotide sequences of the sgRNAs and donor DNA used for the generation of the Pcsk9 reporter cell line.

Supplementary Table 7 Physical characterization of the LNPs used in this study

Supplementary Table 8 List of the primers used in this study.

### Source data

Source Data Fig. 1

Source Data Fig. 2

Source Data Fig. 3

Source Data Fig. 4

Source Data Fig. 5

Source Data Extended Data Fig. 1

Source Data Extended Data Fig. 2

Source Data Extended Data Fig. 3

Source Data Extended Data Fig. 4

Source Data Extended Data Fig. 5

Source Data Extended Data Fig. 6

Source Data Extended Data Fig. 7

## Data Availability

All data are available in the Article or in its Supplementary Information. Data from RNA-seq, WGMS, targeted BS-seq and targeted amplicon sequencing have been deposited in the Gene Expression Omnibus (GEO) database (accession number: GSE226209). Data from RNA-seq, WGMS and targeting sequencing were analysed using the GRCm38 mouse reference genome and the Gencode M25 annotation (https://www.gencodegenes.org/mouse/release_M25.html). Source data are provided with this paper.
