# Supplementary material for: Durable and efficient gene silencing in vivo by hit-and-run epigenome editing
Source: Nature. 2024 Feb 28;627(8003):416–23. doi: 10.1038/s41586-024-07087-8 (PMC10937395; doi:10.1038/s41586-024-07087-8)
Supplement: Supplementary file 1 — Gating strategy for quantification and isolation of Pcsk9tdTomato-negative Hepa 1-6 by flow cytometry [file 41586_2024_7087_MOESM1_ESM.doc]

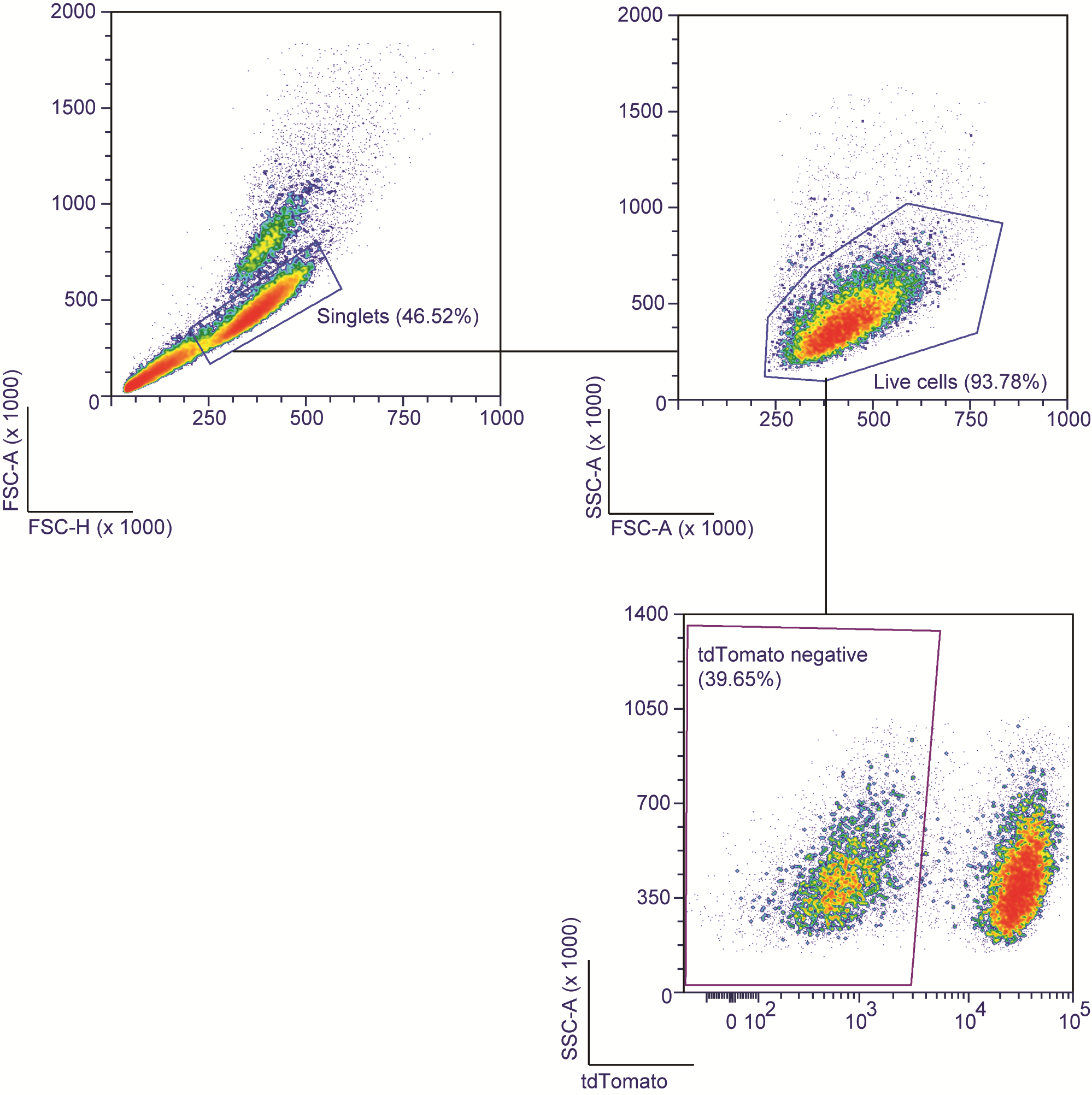


**Supplementary Information Figure 1:** Gating strategy for quantification and isolation of Pcsk9^tdTomato^-negative Hepa 1-6 by flow cytometry. Cells aggregates and debris were excluded by gating cells on the diagonal of FSC-A/FSC-H. Then, viable cells were defined as FSC-high and SSC-low populations. Finally, Pcsk9^tdTomato^-negative Hepa 1-6 was gated.
