# Supplementary material for: Durable and efficient gene silencing in vivo by hit-and-run epigenome editing
Source: Nature. 2024 Feb 28;627(8003):416–23. doi: 10.1038/s41586-024-07087-8 (PMC10937395; doi:10.1038/s41586-024-07087-8)
Supplement: Supplementary file 2 — Reporting Summary [file 41586_2024_7087_MOESM2_ESM.pdf]

nature portfolio

Corresponding author(s): Angelo Lombardo, PhD

Last updated by author(s): Dec 22, 2023

Reporting Summary

Nature Portfolio wishes to improve the reproducibility of the work that we publish. This form provides structure for consistency and transparency in reporting. For further information on Nature Portfolio policies, see our Editorial Policies and the Editorial Policy Checklist.

Statistics

For all statistical analyses, confirm that the following items are present in the figure legend, table legend, main text, or Methods section.

n/a	Confirmed	
☐	☒	The exact sample size (*n*) for each experimental group/condition, given as a discrete number and unit of measurement
☐	☒	A statement on whether measurements were taken from distinct samples or whether the same sample was measured repeatedly
☐	☒	The statistical test(s) used AND whether they are one- or two-sided<br>*Only common tests should be described solely by name; describe more complex techniques in the Methods section.*
☐	☒	A description of all covariates tested
☐	☒	A description of any assumptions or corrections, such as tests of normality and adjustment for multiple comparisons
☐	☒	A full description of the statistical parameters including central tendency (e.g. means) or other basic estimates (e.g. regression coefficient) AND variation (e.g. standard deviation) or associated estimates of uncertainty (e.g. confidence intervals)
☐	☒	For null hypothesis testing, the test statistic (e.g. *F*, *t*, *r*) with confidence intervals, effect sizes, degrees of freedom and *P* value noted<br>*Give P values as exact values whenever suitable.*
☒	☐	For Bayesian analysis, information on the choice of priors and Markov chain Monte Carlo settings
☒	☐	For hierarchical and complex designs, identification of the appropriate level for tests and full reporting of outcomes
☐	☒	Estimates of effect sizes (e.g. Cohen's *d*, Pearson's *r*), indicating how they were calculated

Our web collection on statistics for biologists contains articles on many of the points above.

Software and code

Policy information about availability of computer code

Data collection	CytExpert Software v2.4 (Beckman Coulter);<br>MiSeq Control Software v2.6 (Illumina);<br>NovaSeq Control Software v1.7 (Illumina);<br>HiSeq Control Software v3.4 (Illumina).
Data analysis	FCS express v7 (DeNovo Software);<br>CHOPCHOP v3 (https://chopchop.cbu.uib.no/);<br>Fastqc v0.11.9 (https://www.bioinformatics.babraham.ac.uk/projects/fastqc/);<br>Trim_Galore v0.6.6. (https://www.bioinformatics.babraham.ac.uk/projects/trim_galore/);<br>Bismark read mapper Methylation caller tool v0.23.0;<br>R package MethylKitv1.16.1;<br>STAR v2.7.6a;<br>R package Subread package v2.0.1;<br>GRCm38 murine reference genome and Gencode v M25 annotation (https://www.gencodegenes.org/mouse/release_M25.html);<br>R package sva v3.38.0;<br>R package DESeq2 v1.30.0;<br>R package bsseq v1.26.0;<br>R package DSS v2.44.0;<br>R package ChIPpeakAnno v3.24.2;<br>GraphPad Prism v9 (GraphPad Software);<br>bowtie2 v2.2.5;

BBMap v39.01 (sourceforge.net/projects/bbmap/);
Trimmomatic v0.39 (http://www.usadellab.org/cms/?page=trimmomatic);
CRISPResso2 v2.2.8;
FLASH v1.2.11.

For manuscripts utilizing custom algorithms or software that are central to the research but not yet described in published literature, software must be made available to editors and reviewers. We strongly encourage code deposition in a community repository (e.g. GitHub). See the Nature Portfolio guidelines for submitting code & software for further information.

Data

Policy information about availability of data

All manuscripts must include a data availability statement. This statement should provide the following information, where applicable:

- Accession codes, unique identifiers, or web links for publicly available datasets
- A description of any restrictions on data availability
- For clinical datasets or third party data, please ensure that the statement adheres to our policy

All data are available in the main text, extended data, or supplementary materials. Data from RNA-seq, WGMS, and targeted bisulfite sequencing have been deposited on the Gene Expression Omnibus (GEO) database (accession number: GSE226209). Data from RNA-seq, WGMS, and targeted amplicon sequencing have been analyzed using GRCm38 murine reference genome and Gencode v M25 annotation (https://www.gencodegenes.org/mouse/release_M25.html).

Research involving human participants, their data, or biological material

Policy information about studies with human participants or human data. See also policy information about sex, gender (identity/presentation), and sexual orientation and race, ethnicity and racism.

Reporting on sex and gender	Not applicable to this study
Reporting on race, ethnicity, or other socially relevant groupings	Not applicable to this study
Population characteristics	Not applicable to this study
Recruitment	Not applicable to this study
Ethics oversight	Not applicable to this study

Note that full information on the approval of the study protocol must also be provided in the manuscript.

Field-specific reporting

Please select the one below that is the best fit for your research. If you are not sure, read the appropriate sections before making your selection.

☒ Life sciences ☐ Behavioural & social sciences ☐ Ecological, evolutionary & environmental sciences

For a reference copy of the document with all sections, see nature.com/documents/nr-reporting-summary-flat.pdf

Life sciences study design

All studies must disclose on these points even when the disclosure is negative.

Sample size	No specific sample-size calculation was performed. However, for in vivo studies, we have empirically allocated for each experimental group three to seven animals, to achieve the needed reproducibility. It is worth mentioning here that different experiments including identical conditions produced consistent results, further corroborating the hypothesis that the sample sizes was generally appropriate.
Data exclusions	No data were excluded from the analyses.
Replication	All the in vitro experiments were conducted with technical replicates (n≥2) and the exact number of replicates is indicated in the respective legends. In vivo experiments were designed including multiple mice (n≥3). The exact number of treated animals in any experimental group for any experiment is reported in the figure legends. It is worth mentioning here that, although we did not conduct exact identical and independent experimental replication, we performed multiple experiments using different doses and/or delivery modalities of the very same editors, confirming the reproducibility of all the results included in this work.
Randomization	Upon arrival at the animal house, mice were randomly allocated to different cages. Moreover, after the administration of LNPs, mice from different experimental groups were located in the same cages to minimize eventual confounding factors.
Blinding	No specific blinding strategies were adopted in this study. However, treated and un-treated mice were labelled using progressive numbers. Finally, the operators re-checked the association between label and treatments once the data analysis was completed. A similar strategy was adopted for in vitro studies. Moreover, sample collection, data processing and analysis were often conducted by different operators, thus

further reducing the risk of eventual operator biases.

Reporting for specific materials, systems and methods

We require information from authors about some types of materials, experimental systems and methods used in many studies. Here, indicate whether each material, system or method listed is relevant to your study. If you are not sure if a list item applies to your research, read the appropriate section before selecting a response.

Materials & experimental systems

n/a	Involved in the study
☒	☐ Antibodies
☐	☒ Eukaryotic cell lines
☒	☐ Palaeontology and archaeology
☐	☒ Animals and other organisms
☒	☐ Clinical data
☒	☐ Dual use research of concern
☒	☐ Plants

Methods

n/a	Involved in the study
☒	☐ ChIP-seq
☐	☒ Flow cytometry
☒	☐ MRI-based neuroimaging

Eukaryotic cell lines

Policy information about cell lines and Sex and Gender in Research

Cell line source(s)	Hepa 1-6 (CRL-1830) were purchased from ATCC; primary murine hepatocytes from C57BL/6 male mice were purchased from Biopredic International.
Authentication	The Hepa 1-6 and the primary murine hepatocytes were purchased and used just upon arrival. No authentications were performed.
Mycoplasma contamination	The Hepa 1-6 cell line and its deirivative, as well as the primary murine hepatocytes, were used upon testing negative for Mycoplasma contamination.
Commonly misidentified lines (See ICLAC register)	No misidentfied lines were used in this study.

Animals and other research organisms

Policy information about studies involving animals; ARRIVE guidelines recommended for reporting animal research, and Sex and Gender in Research

Laboratory animals	Eighth week-old C57BL/6N female mice were purchased from Charles River Laboratories (Calco, Italy)
Wild animals	No wild animals were used in this study.
Reporting on sex	Sex was not considered in the study design.
Field-collected samples	The study did not involve samples collected from the filed.
Ethics oversight	Procedures involving animal handling and care followed national and international law and policies and were approved by the Institutional Animal Care and Use Committee (Authorization nos. 604/2020-PR and 233/2022-PR, provided by the Italian Ministry of Health).

Note that full information on the approval of the study protocol must also be provided in the manuscript.

Plants

Seed stocks	Not applicable to this study
Novel plant genotypes	Not applicable to this study
Authentication	Not applicable to this study

Flow Cytometry

Plots

Confirm that:

☒ The axis labels state the marker and fluorochrome used (e.g. CD4-FITC).

☒ The axis scales are clearly visible. Include numbers along axes only for bottom left plot of group (a 'group' is an analysis of identical markers).

☒ All plots are contour plots with outliers or pseudocolor plots.

☒ A numerical value for number of cells or percentage (with statistics) is provided.

Methodology

Sample preparation	Hepa 1-6 were detached using trypsin, blocked in full medium, centrinfuged and resuspended in PBS.
Instrument	CytoFLEX S (Beckman Coulter) and FACSAria™ Fusion Cell Sorter (BD Biosciences).
Software	Flow cytometry was performed using CytoFLEX S (Beckman Coulter) and raw data were analyzed using FCS express (DeNovo Software).
Cell population abundance	Sorted cells: >100.000 cells; purity >95% by CytoFLEX S analysis.
Gating strategy	Cell aggregates and debries were excluded by gaiting cells on the diagonal of FSC-H/FSC-A plot. Then, viable cells were defined as FSC-high and SSC-low population. Wild-type Hepa 1-6 was used to set gates for tdTomato-negative cells.

☒ Tick this box to confirm that a figure exemplifying the gating strategy is provided in the Supplementary Information.
